# Incidence, clinical characteristics, and outcome of interstitial pneumonia in patients with lymphoma

**DOI:** 10.1007/s00277-017-3157-9

**Published:** 2017-10-31

**Authors:** Wei Ping Liu, Xiao Pei Wang, Wen Zheng, Yan Xie, Mei Feng Tu, Ning Jing Lin, Ling Yan Ping, Zhi Tao Ying, Chen Zhang, Li Juan Deng, Ning Ding, Xiao Gan Wang, Yu Qin Song, Jun Zhu

**Affiliations:** Key Laboratory of Carcinogenesis and Translational Research (Ministry of Education), Department of Lymphoma, Peking University Cancer Hospital and Institute, Beijing, China

**Keywords:** Lymphoma, Lung diseases, Interstitial, Therapeutics, Risk factors

## Abstract

Interstitial pneumonia (IP) is a lethal complication in lymphoma patients undergoing chemotherapy. A total of 2212 consecutive patients diagnosed with lymphoma between 2009 and 2014 were enrolled in the present study. IP was defined as diffuse pulmonary interstitial infiltrate found on computed tomography scans. IP was observed in 106 patients. Of these, 23 patients were excluded from the study. Finally, 83 patients with IP were included in this study. The incidence of IP was 3.9% (7/287) in Hodgkin lymphoma and 2.4% (76/1925) in non-Hodgkin lymphoma (*P* = 0.210). The median number of chemotherapy cycles before IP was 3. The median time from the cessation of chemotherapy to IP was 17 days. Eighty-two (98.8%) patients recovered after the treatment with glucocorticoids. Sixty-six (79.5%) patients had a delay in chemotherapy, and 14 (16.9%) patients had premature termination of chemotherapy. Sixty-nine patients were re-treated with chemotherapy after remission from IP, of which 22 (31.9%) experienced IP recurrence. The incidence of IP recurrence was significantly higher in patients re-treated with a similar regimen than in those re-treated with an alternative regimen (65.4 vs. 11.6%, *P* < 0.001). In a multivariate Cox regression analysis, B symptoms and a history of drug allergies were identified as risk factors for IP. In conclusion, IP is a life-threatening complication in lymphoma patients. Glucocorticoid therapy with continuous monitoring of chest radiographic changes may be a favourable strategy for treating IP. However, IP may recur, especially in patients re-treated with a similar chemotherapy regimen.

## Introduction

Interstitial pneumonia (IP) is a heterogeneous disease that includes multiple diffuse parenchymal lung disorders [1, 2]. Several studies have reported IP in lymphoma patients undergoing chemotherapy with or without rituximab [3, 4]. IP may result in dyspnoea, respiratory failure, and death. Additionally, patients who experience IP have more treatment delays and more frequent premature termination of chemotherapy. Overall survival (OS) may be reduced in patients who have recovered from IP. Therefore, treatment of IP is challenging because of its protean, multifaceted nature [5]. Importantly, the incidence of IP recurrence in patients with lymphoma who are re-treated with chemotherapy remains unknown.

In this study, we retrospectively analysed IP in patients with lymphoma. We analysed several features of IP, including the clinical features, recurrence, risk factors, and effects on survival.

## Methods

The study protocol was approved by the Ethics Committee at the Peking University Cancer Hospital and Institute, and the requirement to obtain informed consent was waived.

We searched the database of cancer registries for cases registered between 2009 and 2014 with a diagnosis of lymphoma. Based on follow-up data from the cancer registry system, 2212 patients with lymphoma were enrolled. All clinical information, including demographic findings, clinical presentations, physical examinations, histopathological reports, radiological features, and laboratory results, were investigated. All patients in the study were followed up by medical record review until death or until the last visit at our institute.

**Fig. 1:**
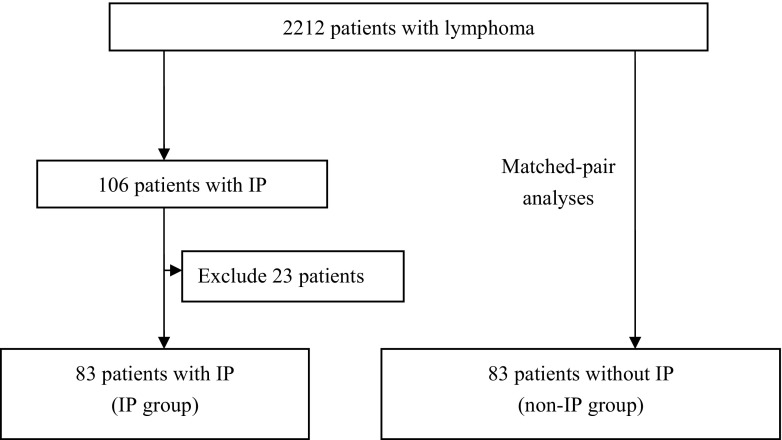
Study design for patients with lymphoma during the study period. (non-IP group: lymphoma patients without IP; IP: interstitial pneumonia)

A glossary of terms compiled by the Fleischner Society [6] was used to assess the thoracic imaging. Based on a previous study [7], IP was defined as follows: diffuse pulmonary interstitial infiltrate identified by computed tomography (CT) in conjunction with respiratory symptoms. IP was identified in 106 patients. To more precisely focus on the association between first-line therapy for lymphoma and IP, 23 patients were excluded for the following reasons: the occurrence of a simultaneous infection (*n* = 6), treatment with a new drug for a clinical trial (*n* = 7), the onset of IP during salvage therapy (*n* = 8), and incomplete documentation (*n* = 2). In total, 83 patients with IP were eligible for this study (Fig. 1). All patients received a chest CT, and 2 (2.4%) patients underwent bronchoalveolar lavage as part of their diagnostic procedure. IP was graded according to the Common Terminology Criteria for Adverse Events (CTCAE version 4.0.3) [8].

We also performed matched-pair analyses. Patients were paired based on whether they had experienced IP, and they were matched for age, sex, and pathological type. Clinical parameters were analysed to identify risk factors for patients with IP.

All statistical analyses were performed using the Statistical Package for the Social Sciences (SPSS version 21.0 for Windows, SPSS, Chicago). Categorical variables were compared using Pearson’s *χ* ^2^ analysis or Fisher’s exact test. Continuous variables were compared using the *t* test or Mann-Whitney rank-sum test. Logistic regression was applied for univariate and multivariate analyses to determine the predictive factors regarding IP. Kaplan-Meier curves were used to compare the differences in progression-free survival (PFS) and OS between the groups, and the log-rank chi-square test was used to calculate the significance of the differences. All statistical tests were two-tailed, and *P* < 0.05 was considered statistically significant.

## Results

### Incidence of IP

The total incidence of IP was 3.75% (83/2212). The incidence of IP was higher in female patients than in male patients (4.8 vs. 3.0%, *P* = 0.031). Although the incidence of IP in Hodgkin lymphoma patients was higher than that in non-Hodgkin lymphoma patients, the difference was not statistically significant (3.9 vs. 2.4%, respectively, *P* = 0.210).

Table 1 summarises the incidence of IP in patients treated with different regimens. In patients with non-Hodgkin lymphoma, the incidence of IP was higher in those treated with the R-CHOP regimen than in those treated with the CHOP regimen (*P* = 0.011). In patients with Hodgkin lymphoma, the incidence of IP was higher in those treated with the BEACOPP regimen than in those treated with the ABVD regimen (*P* < 0.001).

**Table 1:** The incidence of IP in patients treated with different regimens

	Number	IP (%)
R-CHOP-E	14	4 (28.6%)
hyperCVAD	8	1 (12.5%)
CHOP + bleomycin	8	1 (12.5%)
BFM-90	103	8 (7.8%)
BEACOPP	50	3 (6.0%)
R-CHOP	726	43 (5.9%)
R-CVP	53	3 (5.7%)
FCR	25	1 (4.0%)
R-EPOCH	33	1 (3.0%)
CHOP	313	7 (2.2%)
ABVD	217	4 (1.8%)
CHOP-L	119	2 (1.7%)
CHOP-E	131	2 (1.5%)
CHOP-EP	22	1 (1.2%)
CHOP-T	3	0 (0)
COEP	3	0 (0)
COEP-L	40	0 (0)
CVP	25	0 (0)
FC	34	0 (0)
R	24	0 (0)
others	261	3 (1.1%)

*ABVD*, doxorubicin, bleomycin, vincristine, dacarbazine; *BEACOPP*, bleomycin, etoposide, doxorubicin, cyclophosphamide, vincristine, procarbazine, prednisone; *BFM-90*, VDLP (vincristine, daunorubicin, L-asparaginase/polyethylene glycol-conjugated asparaginase, prednisone)/CAT (cyclophosphamide, cytarabine, 6-mercaptopurine)/methotrexate; *CHOP*, cyclophosphamide, doxorubicin, vincristine, prednisone; *COEP*, cyclophosphamide, vincristine, etoposide, prednisone; *CVP*, cyclophosphamide, vincristine, prednisone; *E*, etoposide; *EP*, etoposide, cisplatin; *EPOCH*, etoposide, prednisone, vincristine, cyclophosphamide, doxorubicin; FC: fludarabine, cyclophosphamide; *hyperCVAD*, A (cyclophosphamide, doxorubicin, vincristine, prednisone)/B (methotrexate, cytarabine); *L*, L-asparaginase/polyethylene glycol-conjugated asparaginase; *R*, rituximab; *T*, teniposide

### Baseline characteristics of patients with IP

Of the 83 patients with IP, 39 were males and 44 were females. The mean age of the patients was 55 years (range, 14–81 years). The most frequent pathology type was diffuse large B cell lymphoma (*n* = 41), followed by lymphoblastic lymphoma/leukaemia (*n* = 8), Hodgkin lymphoma (*n* = 7), follicular lymphoma (*n* = 6), mantle cell lymphoma (*n* = 6), marginal zone lymphoma (*n* = 5), Burkitt lymphoma (*n* = 4), angioimmunoblastic T cell lymphoma (*n* = 2), extranodal NK/T cell lymphoma (*n* = 2), chronic lymphocytic leukaemia/small lymphocytic lymphoma (*n* = 1), and anaplastic large cell lymphoma (*n* = 1).

Eight patients were diagnosed at stage I (9.6%), 16 at stage II (19.3%), 9 at stage III (10.8%), and 50 at stage IV (60.3%). The performance status scores of the patients ranged from 0 to 2. Thirty-eight (45.8%) patients presented with B symptoms. Twenty-two (26.5%) patients had a baseline absolute lymphocyte count (ALC) lower than 1 × 10^9^/L. Lung involvement was observed in 9 (10.8%) patients. Thirteen (15.7%) patients had a history of smoking, and 17 (20.5%) patients had a history of drug allergies. No patients received radiotherapy or stem cell transplantation before the onset of IP.

### Clinical features of patients with IP

The median number of chemotherapy cycles prior to the onset of IP was 3 (range, 1–8). The median time from the last treatment to IP was 17 days (range, 1–30 days). Twenty-eight (33.7%) patients were treated with granulocyte colony-stimulating factor (G-CSF) before the emergence of IP.

The severity of pneumonitis was grade 1 in 24 patients (29%), grade 2 in 35 patients (42%), grade 3 in 13 patients (16%), and grade 4 in 11 patients (13%). Most patients diagnosed with IP presented with fever (56.6%), cough (45.8%), and dyspnoea (28.9%). Eleven (13.3%) patients had respiratory failure that was confirmed by arterial blood gas analysis.

On the CT scans, 55 patients presented with acute interstitial pneumonia, 7 had nonspecific interstitial pneumonia, 7 had respiratory bronchiolitis-interstitial lung disease, 5 had cryptogenic organising pneumonia, 4 had desquamative interstitial pneumonia, 3 had lymphoid interstitial pneumonia, and 2 had usual interstitial pneumonia.

### Treatment and outcome

Glucocorticoids were provided for all patients. No patients required mechanical ventilation. Among 11 patients with respiratory failure, 9 were treated with intravenous methylprednisolone (1–1.5 mg/kg/day) and 2 were treated with oral prednisone (1–1.5 mg/kg/day). Among 72 patients without respiratory failure, 58 were treated with intravenous methylprednisolone (0.5–1 mg/kg/day), 12 were treated with oral prednisone (0.25–1 mg/kg/day), and 2 were treated with intravenous dexamethasone (0.1 mg/kg/day). The dose of glucocorticoids was gradually reduced by 25% every week if the remission of IP was confirmed by CT.

Anti-microorganism drugs, including 4-quinolones (26.5%), sulfamethoxazole-trimethoprim (16.9%), carbapenems (7.2%), cephalosporins (4.8%), ganciclovir (6.0%), and antifungals (3.6%), were administered to 44 (53.0%) patients despite a lack of evidence of infection.

Eighty-two (98.8%) patients recovered from the IP episodes, and 1 (1.2%) patient with acute interstitial pneumonia died. The median time to remission was 24 days (range, 3–75 days). The median time to remission of IP for patients with or without respiratory failure was 34 or 22 days, respectively (*P* = 0.022). The median time to remission of IP for patients treated with or without prophylactic anti-microorganism drugs was 20 or 28 days, respectively (*P* = 0.111).

### IP recurrence

Sixty-nine (83.1%) patients were re-treated with chemotherapy after remission of IP. Of those, 26 patients were re-treated with a regimen that was similar to the previous chemotherapy treatment, and 43 patients were re-treated with an alternative regimen. In total, 22 (31.9%) patients experienced IP recurrence. The incidence of IP recurrence for patients re-treated with a similar or an alternative regimen was 65.4 or 11.6%, respectively (χ^2^ = 21.559, *P* < 0.001).

Glucocorticoids were provided for all patients with IP recurrence. Twenty-two (90.9%) patients recovered from the IP episodes, and 2 (9.1%) patients with acute interstitial pneumonia died.

### Risk factors for IP

To investigate the risk factors of IP, matched-pair analyses were performed. The clinical parameters are summarised in Table 2. Compared to patients without IP, more patients with IP had advanced stage, B symptoms, and a history of drug allergies, and fewer patients with IP had a history of smoking.

**Table 2:** Comparison of clinical parameters between patients with and without IP

	Patients with IP (%)	Patients without IP (%)	*P*
Advanced stage	59 (71.1%)	43 (51.8%)	0.011
B symptom	38 (45.8%)	17 (20.5%)	0.001
Lung involvement	9 (10.8%)	7 (8.4%)	0.599
History of drug allergy	17 (20.5%)	7 (8.4%)	0.027
History of cigarette smoking	13 (15.7%)	26 (31.3%)	0.017
Diabetes mellitus	8 (9.6%)	8 (9.6%)	1.0
Autoimmune disease	3 (3.6%)	2 (2.4%)	0.650
ECOG > 1	6 (7.2%)	1 (1.2%)	0.053
Extranodal involvement	65 (78.3%)	56 (67.5%)	0.116
Rituximab	56 (67.5%)	47 (56.6%)	0.150
Bleomycin	7 (8.4%)	7 (8.4%)	1.000
ALC < 1 × 10^9^/L	20 (24.1%)	15 (18.1%)	0.341

*ALC*, absolute lymphocyte count; *ECOG*, Eastern Cooperative Oncology Group; *IP*, interstitial pneumonia

The univariate analysis revealed that age, advanced stage, B symptoms, a history of drug allergies, and a history of smoking were statistically significant risk factors for IP (Table 3). In the multivariate Cox regression analysis, B symptoms and a history of drug allergies were identified as risk factors of IP.

**Table 3:** Analysis of risk factors for interstitial pneumonia

	Univariate logistic analysis			Multivariate Cox analysis		
	OR	95%CI	*P*	OR	95%CI	*P*
Advanced stage	2.287	1.205–4.340	0.011			
B symptom	3.278	1.651–6.510	0.001	4.221	1.852–9.622	0.001
Lung involvement	1.320	0.468–3.730	0.600			
History of drug allergy	2.797	1.092–7.159	0.032	4.019	1.375–11.750	0.011
History of cigarette smoking	0.407	0.192–0.864	0.019			
ECOG > 1	1.613	0.940–2.768	0.083			
Extranodal involvement	1.741	0.869–3.490	0.118			
Rituximab	1.589	0.844–2.989	0.151			
Bleomycin	1.000	0.335–2.989	1.000			
ALC < 1 × 10^9^/L	1.439	0.678–3.053	0.343			

*ALC*, absolute lymphocyte count; *CI*, confidence interval; *ECOG*, Eastern Cooperative Oncology Group; *OR*, odds ratio

### Impact of IP on chemotherapy and survival

Eight (96.4%) patients experienced discontinuous chemotherapy. Of those, 66 (79.5%) patients had chemotherapy delays, and 14 (16.9%) patients had premature termination of chemotherapy.

After 32.07 months of follow-up, disease progression was observed in 36 (43.4%) patients with IP and in 22 (26.5%) patients without IP (*χ* ^2^ = 5.194, *P* = 0.023). The expected 5-year PFS rates were 40 and 67% for those with and without IP, respectively (*P* = 0.006).

At the end of the study, 31 (37.3%) patients with IP and 16 (19.3%) patients without IP had died. The expected 5-year OS rates were 52 and 75% for patients with and without IP, respectively (*P* = 0.006).

## Discussion

IP is not rare and might be a substantial comorbidity in patients with lymphoma. Salmasi et al. [9] found that the incidence of IP was 2.9% in 560 patients with B cell lymphoma, and a higher rate was observed in the rituximab group (3.95 vs. 1.3%). Huang et al. [7] reported that the total incidence of IP was 4.9% in 529 patients with diffuse large B cell lymphoma, and the addition of rituximab to the chemotherapy regimen was identified as an independent risk factor for IP. In our study, the total incidence of IP among 2212 patients with lymphoma was 3.75%, and a higher incidence of IP was observed in female patients than in male patients. Importantly, the incidence of IP was different in patients treated with different regimens. Consistent with previous studies [3, 7, 9], the incidence of IP was higher in those treated with the R-CHOP regimen than in those treated with the CHOP regimen.

The diagnosis of drug-induced IP is made based on the clinical symptoms, physical findings, history of drug use and diseases, and diagnostic imaging and pathological findings taken as a whole [10]. When IP is suspected, CT and laboratory tests such as blood cell count and bacterial culture should be performed for differential diagnosis, followed by bronchoalveolar lavage fluid analysis and lung biopsy. Infection plays an important role in the pathogenesis of IP [11] and should be carefully ruled out before diagnosis of drug-induced IP. Although 6 patients were excluded because of a simultaneous infection with confirmed pathogenic evidence in our study, only 2 of 83 patients underwent bronchoalveolar lavage as part of their diagnostic procedure. As a result, infection could not be completely ruled out in cases suspected of drug-induced IP. Moreover, prophylactic anti-microorganism drugs were administered to 53.0% of the patients, which might have interfered with the results of microbiological culture.

The early diagnosis and management of IP present challenges for the physician, because the spectrum of severity of IP ranges from mild dyspnoea to fatal pulmonary disorders such as respiratory failure. Park et al. [12] reported that interstitial pneumonitis accounted for 54.8% of non-neutropenic fevers, and the causative organism was not identified in the majority of cases. In our study, grade 1–2 pneumonitis was observed in 71% of patients according to CTCAT 4.03. Fever was the most frequent clinical manifestation of IP, followed by non-productive cough and dyspnoea. Notably, 11 patients had grade 4 pneumonitis because they experienced respiratory failure at onset, which suggested that those patients with IP should be monitored carefully and promptly diagnosed and treated.

Based on the severity of IP, distinct treatment approaches are required [13]. Interruption of chemotherapy should be considered for those patients with any symptoms. Early interventional use of glucocorticosteroids for IP is recommended in different guidelines [14, 15], because it can suppress alveolar protein leak and reduce the severity of the inflammatory cell response [16–18]. Low-dose steroids are recommended (e.g. prednisone 1–2 mg/kg/day PO or methylprednisolone 1–2 mg/kg/day IV) for grade 2 pneumonitis, and high-dose steroids with methylprednisolone (e.g. 1 g/day IV) was for grade 3 pneumonitis [13]. However, the optimal dose and course of glucocorticoids remain unclear [19], and prospective studies integrated with a schedule for follow-up investigation of the subtype of IP could be helpful in addressing this. In our study, intravenous or oral glucocorticoids at an initial dose of 0.5–1.5 mg/kg/day were administered to most patients and were tapered gradually (reduced by 25% every week) in those patients who were responding. Given that the continuous use of steroids may significantly increase the risk of pulmonary infections, including *Pneumocystis jiroveci* pneumonia [20], anti-microorganism drugs were administered to more than half of the patients. Except for 1 patient who died of IP progression, the remaining 82 (98.8%) patients recovered from the IP episodes with a median time to remission of 24 days. This finding revealed that glucocorticoid therapy with continuous monitoring of chest radiographic changes may be a favourable treatment strategy for IP.

Some preliminary evidence suggested that antitumour therapy may be continued for a life-threatening flare of tumour growth under steroid cover and/or at a reduced dose [21]. However, re-treatment with antitumour agents presents a clinical dilemma due to the risk of IP recurrence. Therefore, this approach requires a careful individualised risk-benefit analysis. In a case series including 9 patients with IP undergoing rituximab-containing chemotherapy, Liu et al. [22] reported that a recurrence of IP was observed in 2 of the 4 lymphoma patients re-treated with rituximab. High morbidity and mortality of IP recurrence were observed in our study. IP recurrence was observed in 31.9% of patients re-treated with chemotherapy, and the incidence of IP recurrence in those patients re-treated with a similar regimen was significantly higher than in those re-treated with an alternative regimen (65.4 vs. 11.6%, respectively). Although glucocorticoids were provided promptly, a high mortality rate of 9.1% was observed. Therefore, re-initiation of a similar regimen should be avoided after remission of IP, and switching to an available alternative regimen should be considered if chemotherapy is resumed.

2Those lymphoma patients with IP had poor prognosis; some patients died of IP itself or from tumour progression after discontinuation of treatment. A retrospective study [23] including 141 patients with Hodgkin lymphoma reported a decrease in OS from 90% in unaffected patients to 63% in patients with bleomycin pulmonary toxicity. In our study, 96.4% of patients experienced chemotherapy delays or premature termination of chemotherapy, and increased disease progression, increased deaths due to tumours, and decreased OS were observed in these patients. Therefore, aggressive management of IP in lymphoma patients is strongly advised, and further prospective studies are warranted.

Due to inconsistent pathological types and treatment regimens, several studies have suggested that the use of rituximab, bleomycin, and G-CSF as well as low absolute lymphocyte count is a risk factor for IP [7, 23, 24]. Moreover, newly introduced targeted drugs for lymphoma, such as idelalisib [25], ibrutinib [26], anti-CD30 monoclonal antibody [27], and anti-PD1 monoclonal antibody [28], have been shown to be associated with drug-induced pneumonitis, making it more difficult to identify risk factors for IP. Lee et al. [29] reported that female gender was a risk factor for non-infectious interstitial lung disease in 340 patients following autologous haematopoietic stem cell transplantation. Similarly, our study demonstrated that female patients had a higher incidence of IP than male patients. To minimise the impact of confounding factors, a matched-pair analysis was performed. History of cigarette smoking seemed to be associated with a decreased risk for IP in univariate logistic analysis, but this correlation was not seen in multivariate Cox analysis. Of note was that B symptoms and a history of drug allergies were identified as risk factors for IP in our study. These findings might help make clinicians more aware of this severe condition and refine the parameters used to identify individuals at higher risk for IP. Properly designed prospective studies should still be performed for validation.

There are several limitations that should be considered when interpreting the results of the present study. First, due to the retrospective nature of this study, it is difficult to completely rule out other causes of IP in our study. Second, a large number of combined chemotherapy regimens using different drugs were employed, and therefore, the impact of specific drugs on IP could not be analysed. Finally, although the risk factors for IP were identified in a match-paired analysis, the small sample size should also be taken into account.

In conclusion, based on a large dataset, we estimated the incidence of IP in lymphoma patients receiving first-line therapy and demonstrated the disappointing clinical outcomes of this adverse event, with considerable associated mortality after IP recurrence. Clinical oncologists should be aware of the recurrence of IP in patients re-treated with antitumour antigens after IP recovery.
